# Deficiency of Myeloid Pfkfb3 Protects Mice From Lung Edema and Cardiac Dysfunction in LPS-Induced Endotoxemia

**DOI:** 10.3389/fcvm.2021.745810

**Published:** 2021-09-29

**Authors:** Jiean Xu, Lina Wang, Qiuhua Yang, Qian Ma, Yaqi Zhou, Yongfeng Cai, Xiaoxiao Mao, Qingen Da, Tammy Lu, Yunchao Su, Zsolt Bagi, Rudolf Lucas, Zhiping Liu, Mei Hong, Kunfu Ouyang, Yuqing Huo

**Affiliations:** ^1^State Key Laboratory of Chemical Oncogenomics, Key Laboratory of Chemical Genomics, School of Chemical Biology and Biotechnology, Peking University Shenzhen Graduate School, Shenzhen, China; ^2^Department of Cellular Biology and Anatomy, Vascular Biology Center, Medical College of Georgia, Augusta University, Augusta, GA, United States; ^3^Department of Cardiovascular Surgery, Peking University Shenzhen Hospital, Shenzhen, China; ^4^Oxford College, Emory University, Oxford, GA, United States; ^5^Department of Pharmacology & Toxicology, Medical College of Georgia, Augusta University, Augusta, GA, United States; ^6^Department of Physiology, Medical College of Georgia, Augusta University, Augusta, GA, United States; ^7^College of Pharmacy, Jinan University, Guangzhou, China

**Keywords:** inflammation, glycolysis, PFKFB3, macrophage, endotoxemia

## Abstract

Sepsis, a pathology resulting from excessive inflammatory response that leads to multiple organ failure, is a major cause of mortality in intensive care units. Macrophages play an important role in the pathophysiology of sepsis. Accumulating evidence has suggested an upregulated rate of aerobic glycolysis as a key common feature of activated proinflammatory macrophages. Here, we identified a crucial role of myeloid 6-phosphofructo-2-kinase/fructose-2,6-bisphosphatase 3 (Pfkfb3), a glycolytic activator in lipopolysaccharide (LPS)-induced endotoxemia in mice. Pfkfb3 expression is substantially increased in bone marrow derived macrophages (BMDMs) treated with LPS *in vitro* and in lung macrophages of mice challenged with LPS *in vivo*. Myeloid-specific knockout of *Pfkfb3* in mice protects against LPS-induced lung edema, cardiac dysfunction and hypotension, which were associated with decreased expression of interleukin 1 beta (Il1b), interleukin 6 (Il6) and nitric oxide synthase 2 (Nos2), as well as reduced infiltration of neutrophils and macrophages in lung tissue. Pfkfb3 ablation in cultured macrophages attenuated LPS-induced glycolytic flux, resulting in a decrease in proinflammatory gene expression. Mechanistically, Pfkfb3 ablation or inhibition with a Pfkfb3 inhibitor AZ26 suppresses LPS-induced proinflammatory gene expression via the NF-κB signaling pathway. In summary, our study reveals the critical role of Pfkfb3 in LPS-induced sepsis *via* reprogramming macrophage metabolism and regulating proinflammatory gene expression. Therefore, PFKFB3 is a potential target for the prevention and treatment of inflammatory diseases such as sepsis.

## Introduction

Sepsis is characterized as a life-threatening organ dysfunction caused by a dysregulated host response to an infectious organism (1), including the new virus SARS-CoV-2 responsible for the COVID-19 ongoing pandemic in humans (2). Infectious organisms stimulate the release of inflammatory cytokines such as interleukin 1 beta (IL1B) and interleukin 6 (IL6), and inducible nitric oxide synthase 2 (NOS2) (3). High levels of these inflammatory cytokines and overproduction of nitric oxide (NO) generated by NOS2 cause severe hypotension and multiple organ dysfunction, eventually leading to death. Although great advances have been made in antibiotics and supportive care, sepsis still remains a major cause of morbidity and mortality in the intensive care units (4). Therefore, there is an urgent need to increase our understanding of the mechanisms involved in the pathogenesis of sepsis for development of new therapeutic targets (5).

Compared to the previous definition of sepsis in 1991 (Sepsis 1.0) and 2001 (Sepsis 2.0), “lactate > 2 mmol/L” was added in the latest definition of sepsis shock in 2016 (Sepsis 3.0) (6), highlighting that glucose metabolism disorders are an important pathogenesis of sepsis. Macrophages, important cells of the innate immune system, play an essential role in the hyper-inflammation stage in sepsis (7). Previous evidence suggests that macrophages display different functional phenotypes depending on their metabolic profiles (8, 9). Pathogen-stimulated macrophages shift their metabolic profiles from oxidative phosphorylation to aerobic glycolysis, coupled with increased secretion of lactate and proinflammatory cytokines, and ultimately supporting the hyper-inflammatory state during sepsis. Data from genome-wide gene expression profiles show that *Pfkfb3* (6-phosphofructo-2-kinase/fructose-2,6-bisphosphatase 3) is the most upregulated gene among the glycolytic genes in LPS-stimulated macrophages (10), indicating a potential role of PFKFB3 in LPS-induced sepsis. PFKFB3 is a bifunctional enzyme that catalyzes the synthesis and hydrolysis of fructose-2,6-bisphosphate (F-2,6-BP). F-2,6-BP acts as the most effective allosteric activator of 6-phosphofructo-1-kinase (PFK1), the second rate-limiting enzyme in glycolysis (11, 12). A previous study demonstrated that zinc fingers and homeoboxes (Zhx) 2 accelerates sepsis by promoting macrophage glycolysis *via* Pfkfb3 (13). However, it remains unclear whether decreased macrophage glycolysis *via* Pfkfb3 inactivation can suppress macrophage inflammation and protect mice from LPS-induced sepsis.

In the current study, we identified a causative role of macrophage Pfkfb3-dependent glycolysis in LPS-induced sepsis through regulation of macrophage inflammation. Myeloid-specific *Pfkfb3* deletion protected mice against LPS-induced lung edema, cardiac dysfunction and inflammation. Mechanistically, Pfkfb3 inactivation in macrophages suppresses LPS-induced inflammation *via* inhibiting the NF-κB signaling pathway. Our findings here expand our understanding of glycolytic regulation of sepsis and indicate PFKFB3 as an attractive potential therapeutic target for the prevention and treatment of sepsis.

## Materials and Methods

### Animals

Mice were used in accordance with the protocol approved by the Institutional Animal Care and Use Committee of Augusta University. The floxed *Pfkfb3* (*Pfkfb3*^flox/flox^) mice were generated by Xenogen Biosciences Corporation (Cranbury, NJ, USA) (14). Myeloid-specific *Pfkfb3* knockout was achieved by cross-breeding *Pfkfb3*^flox/flox^ mice with *Lysm*-Cre mice (stock no. 004781, The Jackson Laboratory, Bar Harbor, ME, USA) to generate *Pfkfb3*^flox/flox^; *Lysm*-Cre (*Pfkfb3*^ΔMϕ^) mice. All mice were on a C57BL/6J background.

### LPS-Induced Sepsis Model

For LPS-induced sepsis, age (8- to 12-week-old)- and sex-matched *Pfkfb3*^ΔMϕ^ and *Pfkfb3*^WT^ mice were administered with LPS (L2630, Sigma-Aldrich, St. Louis, MO, USA) at 12.5 mg/kg body weight by intraperitoneal injection. The mice had normal access to food and water and were monitored twice a day over the course of 10 days. The rectal temperature of mice was measured with a model BAT-12 digital thermocouple thermometer (Physitemp Instruments, Clifton, NJ, USA) before and 6 h, 24 h after LPS injection.

### Blood Pressure Measurements

Mouse blood pressure (BP) was measured at the same time of day using a noninvasive tail-cuff blood pressure measurement system (Coda 6, Kent Scientific, Torrington, CT, USA). In brief, mice were trained for BP measurement conditions on a daily basis for 1 week. After training, mouse BP was measured twice before the injection of LPS and at indicated times following LPS injection. For BP measurements, we placed conscious mice in tail-cuff restrainers over a warmed surface. Twenty consecutive BP measurements were taken, and the last fifteen readings per mouse were averaged and used for analysis.

### Ultrasound Imaging

Echocardiograms were performed in isoflurane-anesthetized mice using the Vevo 2100 high-frequency ultrasound imaging platform (FUJIFILM VisualSonics, Toronto, Ontario, Canada) at baseline and 6 h after LPS injection. After sedation, mice were laid on the heating platform (37 °C) to maintain normothermia and continuously delivered a gas inhalation of two percent isoflurane. The prewarmed ultrasonic gel (4238, Chattanooga, Vista, CA, USA) was added to the mouse's chest after removal of hair with depilatory cream. Two-dimensional echocardiography was performed with a 40 MHz ultrasound probe (MS-400). The percentage of left ventricular (LV) ejection fraction and fractional shortening, LV stroke volume and cardiac output were obtained from the parasternal short axis view using M-mode.

### Pulmonary Permeability Assessment

Lung wet-to-dry weight ratio was used as an index of pulmonary edema formation that served as a gauge for measuring pulmonary permeability. The lung was weighed immediately after its excision (wet weight), and then placed into an oven at 60 °C for 48 h, and reweighed as dry weight. The ratio of the lung weight before and after drying was calculated.

Pulmonary permeability was also evaluated with Evans Blue dye. Briefly, LPS (1 mg/kg body weight) or saline was intratracheally injected to mice via a 20-gauge catheter. In order to assess vascular leak, Evans blue (30 mg/kg body weight, E2129, Sigma-Aldrich, St. Louis, MO, USA), was injected to mice via the tail vein 2 h before mice were sacrificed. The lungs were perfused with PBS, then dried with tissue papers, and left lung were imaged.

### BMDM Culture and Treatments

After euthanization of mice, femurs and tibias were isolated and transected. Bone marrow cells were flushed from the femurs and tibias. The cell suspension was pipetted repeatedly to obtain a single-cell suspension, which was then filtered with a 70 μm cell strainer and centrifuged at 500 g for 8 min. The acquired cells were plated at a density of 2 × 10^6^/mL and cultured in RPMI 1640 medium (SH30027.01, Cytiva, Marlborough, MA, USA) supplemented with 10% FBS (F4135, Sigma-Aldrich, St. Louis, MO, USA), 20% L929-conditioned medium, and 1 × Antibiotic-Antimycotic (15240062, Thermo Scientific, Grand Island, NY, USA) in a humidified incubator with 5% CO_2_ at 37°C for 6 days to induce macrophage differentiation. In some experiments, cells were incubated with 100 ng/mL LPS (L3137, Sigma-Aldrich, St. Louis, MO, USA) or 10 μm AZ26 (HY-101971, MedChemExpress, Monmouth Junction, NJ, USA) at various time points as indicated.

### Measurement of Cytokines by ELISA

Serum samples were collected using a standard protocol following intraperitoneal injection with PBS (control) or LPS (12.5 mg/kg body weight) into *Pfkfb3*^WT^ and *Pfkfb3*^ΔMϕ^ mice for 6 h. Cell culture supernatants were collected from *Pfkfb3*^WT^ and *Pfkfb3*^ΔMϕ^ BMDMs treated with LPS for 16 h (100 ng/mL). Cell culture supernatants and sera were measured for mouse Il1b and Il6 with ELISA kits (Il1b, MLB00C; Il6, M6000B, R&D Systems, Minneapolis, MN, USA). All assays were carried out following the instructions provided by the manufacturer.

### Analysis of Nitric Oxide (NO) Release

NO release was measured using a Sievers NOA 280i chemiluminescence analyzer (Analytix, Sunderland, UK) as previously described (15). Briefly, 20 μl of supernatant or 100 μL of serum was injected into a nitrogen-purge vessel containing a 1% solution of sodium iodide in glacial acetic acid. The output was recorded using a Labchart program (ADInstruments, Colorado Springs, CO, USA) and the area under the curve was converted to picomole NO using a calibration curve constructed after the analysis of a series of sodium nitrite standards ranging from 2.5 to 100 pmol.

### Real Time Cell Metabolism Assay

Real-time changes in extracellular acidification rate (ECAR) of BMDMs were analyzed with an XF24 Extracellular Flux Analyzer as described previously (16). Briefly, BMDMs were seeded at 2 × 10^5^ per well onto Seahorse XF24 culture microplates (100777-004, Seahorse Bioscience, North Billerica, MA, USA) and incubated in a humidified incubator with 5% CO_2_ at 37°C overnight. The next day, after 100 ng/mL LPS treatment for 6 h, the medium was changed to a XF base Medium (102353-100, Seahorse Bioscience, North Billerica, MA, USA) supplemented with 2 mM glutamine, pH adjusted to 7.4 with 0.1 M NaOH, and then the plate was incubated for 45 min in a non-CO_2_ incubator at 37°C. The ECAR assay was performed on the XF24 extracellular flux analyzer (Seahorse Bioscience, North Billerica, MA, USA), and the ECAR values were normalized using protein concentration. Inhibitors and activators were used in these tests at the following concentrations: glucose (10 mM), oligomycin (2 μM) and 2-DG (50 mM).

### Real-Time Quantitative PCR (RT-qPCR) Analysis

Total RNA of cells and tissues were extracted using TRIzol reagent (15596018, Invitrogen, Grand Island, NY, USA) according to the manufacturer's instructions, and RT-qPCR was done as described previously (17). Briefly, one microgram of total RNA was used to synthesize first stranded cDNA with the iScript^TM^ cDNA synthesis kit (1708891, Bio-Rad, Hercules, CA, USA). RT-qPCR was performed on a QuantStudio^TM^ 3 Real-Time PCR System (Applied Biosystems, Grand Island, NY, USA) using iTaq^TM^ Universal SYBR Green Supermix (1725122, Bio-Rad, Hercules, CA, USA) with the respective gene-specific primers listed in Supplementary Table 1. Quantification of relative gene expression was calculated with the 2^−ΔΔCt^ method using *18S* rRNA as the internal control, and data were expressed as fold change relative to control groups.

### Western-Blot

Western blot was performed as previously described (18). Cells and tissues were homogenized in RIPA buffer (R0278, Sigma-Aldrich, St. Louis, MO, USA) supplemented with 1% protease inhibitor cocktail (05892970001, Sigma-Aldrich, St. Louis, MO, USA) and 1% phosphatase inhibitor (4906845001, Sigma-Aldrich, St. Louis, MO, USA). After measuring protein concentration using BCA Protein Assay Kit (23225, Thermo Scientific, Grand Island, NY, USA), equal amounts of denatured proteins were loaded and separated onto a 7.5–10% SDS-PAGE gel, and transferred on nitrocellulose membranes (10600015, Cytiva, Marlborough, MA, USA). Membranes were blocked with 5% non-fat milk, then incubated with specific antibodies. The antibodies used were as follows: rabbit Pfkfb3 (1:1000, ab181861, Abcam, Cambridge, MA, USA), rabbit Nos2 (1:1000, sc-650, Santa Cruz, Dallas, TX, USA), rabbit p-Erk (1:1000, 4370S, Cell Signaling Technology, Danvers, MA, USA), rabbit Erk (1:1000, 4695S, Cell Signaling Technology, Danvers, MA, USA), rabbit p-Jnk (1:1000, 4668S, Cell Signaling Technology, Danvers, MA, USA), rabbit Jnk (1:1000, 9252S, Cell Signaling Technology, Danvers, MA, USA), rabbit p-p38 (1:1000, 9215S, Cell Signaling Technology, Danvers, MA, USA), rabbit p38 (1:1000, 9212S, Cell Signaling Technology, Danvers, MA, USA), rabbit p-p65 (1:1000, 3033S, Cell Signaling Technology, Danvers, MA, USA), rabbit p65 (1:1000, 8242S, Cell Signaling Technology, Danvers, MA, USA), and mouse Actb (1:1000, sc-47778, Santa Cruz, Dallas, TX, USA). Images were taken with the ChemiDoc Imaging System (Bio-Rad, Hercules, CA, USA), and band densities were identified using ImageJ (National Institutes of Health, Bethesda, MD, USA).

### Histological Analysis

Murine lungs were flushed with PBS and 4% PFA, fixed and embedded in paraffin according to a standard protocol. Five micrometer paraffin-embedded sections were cut and stained with hematoxylin and eosin (H&E) routinely, the lung injury score was assessed based on the method as reported previously (19). Immunohistochemical staining was performed as described previously (20), briefly, lung sections were first deparaffinized and rehydrated, endogenous peroxidase activity was destroyed with H_2_O_2_ (3 mL 30% H_2_O_2_ in 200 mL methanol) for 30 min at room temperature. After antigen retrieval with Antigen Unmasking Solution (H-3301, Vector Laboratories, Burlingame, CA, USA) at 98°C for 10 min, sections were blocked with avidin solution with 10% normal rabbit serum for 1 h at room temperature, and incubated in biotin blocking solution with primary antibodies against Mac2 (3 μg/mL, CL8942F, Cedarlane, Burlington, NC, USA), or Ly6G (3 μg/mL, 551459, BD biosciences, San Jose, CA, USA) at 4°C overnight. Sections were then incubated with a biotinylated rabbit anti-rat IgG secondary antibody (1:200, BA-4001-0.5, Vector Laboratories, Burlingame, CA, USA) for 1 h at room temperature, followed by incubation with ABC solution (PK-6100, Vector Laboratories, Burlingame, CA, USA) for 30 min at room temperature. Peroxidase substrate 3, 3′-diaminobenzidine (3468, Dako, Santa Clara, CA, USA) was used to detect the antibodies according to the manufacturer's instructions. The sections were counterstained with hematoxylin for 30 sec, then dehydrated and mounted with xylene-based mounting medium (8312-4, Thermo Scientific, Grand Island, NY, USA). Quantification of Mac2 and Ly6G staining was performed using the ImageJ (National Institutes of Health, Bethesda, MD, USA).

For immunofluorescence staining of lung sections, 5 μm paraffin-embedded sections were deparaffinized and rehydrated. After antigen retrieval with Antigen Unmasking Solution (H-3301, Vector Laboratories, Burlingame, CA, USA) at 98°C for 10 min, sections were blocked and incubated at 4°C overnight with primary antibodies against Pfkfb3 (1:100, ab181861, Abcam, Cambridge, MA, USA) and Adgre1 (1:100, ab6640, Abcam, Cambridge, MA, USA). For immunofluorescence staining of BMDMs, cells seeded on culture slides (354108, Corning, Glendale, AZ, USA) were fixed in 4% PFA for 20 min, permeabilized with 0.5% Triton X-100 for 20 min, and then blocked and incubated at 4°C overnight with primary antibodies against Pfkfb3 (1:100, ab181861, Abcam, Cambridge, MA, USA) and Adgre1 (1:100, MCA497R, Bio-Rad, Hercules, CA, USA). Sections and slices were incubated at room temperature for 1 h with Alexa Fluor 594-labeled goat anti-rabbit IgG (8 μg/mL, A11012, Molecular Probes, Grand Island, NY, USA) and Alexa Fluor 488-labeled goat anti-rat IgG (8 μg/mL, A11006, Molecular Probes, Grand Island, NY, USA), stained with 4′,6-diamidino-2-phenylindole (1 μg/mL, D1306, Invitrogen, Grand Island, NY, USA) for 5 min, and immersed in mounting medium (H-1000, Vector Laboratories, Burlingame, CA, USA). Images were acquired with a confocal microscope (Zeiss 780 Upright Confocal, Carl Zeiss, Oberkochen, Germany), and analyzed using ImageJ (National Institutes of Health, Bethesda, MD, USA).

### Statistical Analysis

Statistical analyses were performed with GraphPad Prism 9 software (GraphPad, La Jolla, CA, USA). Categorical data are shown as percentages, and Mantel-Cox test was used to compare differences between the two groups (survival curves). Continuous variables are presented as the means ± SEM, two-tailed unpaired Student's *t*-test was used to compare the difference between two groups, and one-way analysis of variance (ANOVA) followed by Bonferroni's *post-hoc* test was performed for comparison among more than two groups when appropriate. Differences were considered statistically significant at *P* < 0.05 (^*^P < 0.05, ^**^P < 0.01 and ^***^P < 0.001).

## Results

### The Expression of Pfkfb3 Is Upregulated in LPS-Stimulated Macrophages

**Figure 1:**
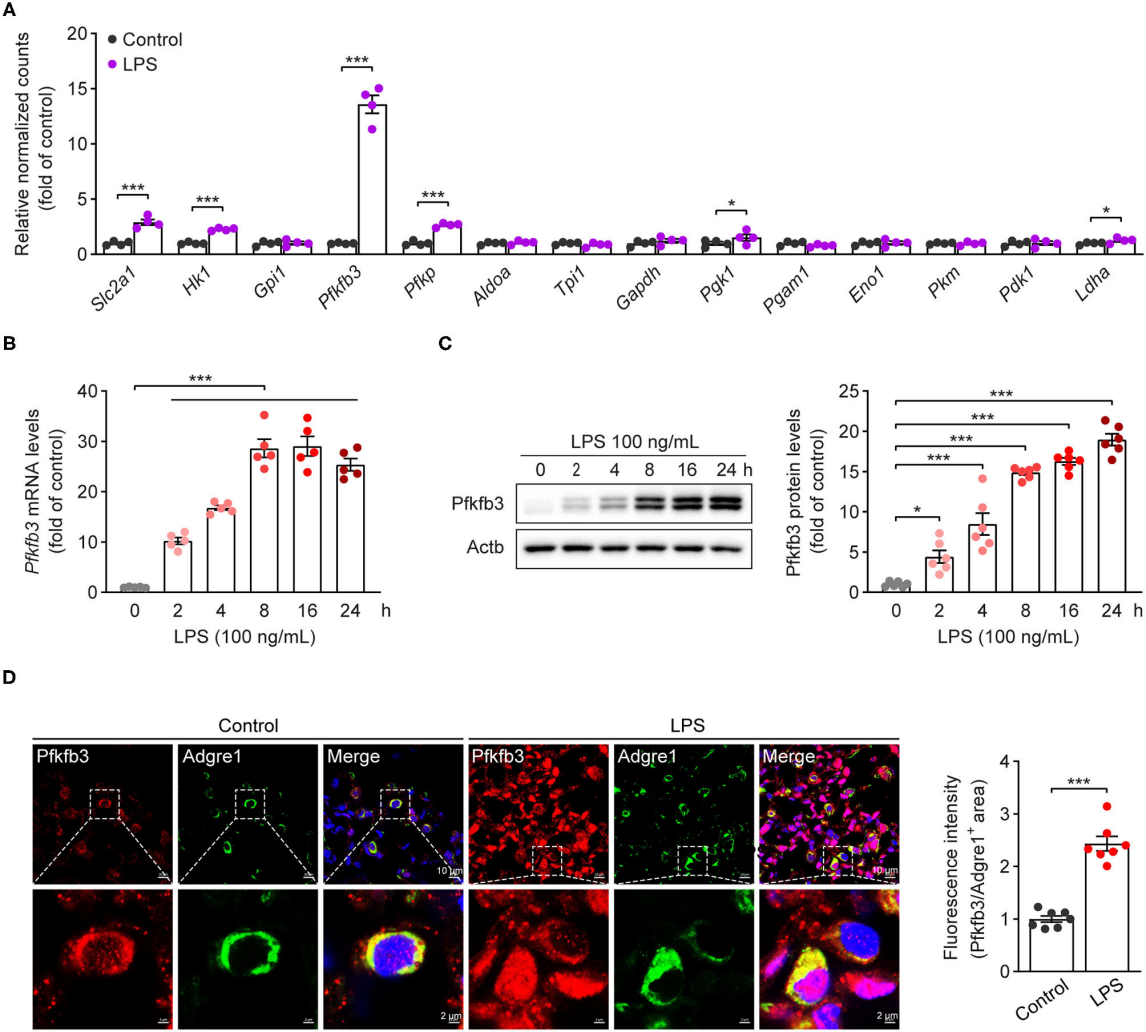
Pfkfb3 expression is upregulated in macrophages treated with LPS. **(A)** The expression of glycolytic genes in BMDMs treated with LPS (100 ng/mL) for 4 h, the data are genome mRNA expression profiling data (GSE125036) from the GEO database (*n* = 4). **(B)** qPCR analysis of *Pfkfb3* expression in BMDMs treated with LPS (100 ng/mL) for the indicated times (*n* = 5). **(C)** Representative Western blot results of Pfkfb3 and Actb in BMDMs treated with LPS (100 ng/mL) for the indicated times (left) and relative ratio of Pfkfb3/Actb (right) were quantitated by densitometric analysis of the corresponding Western blots (*n* = 6). **(D)** Representative images (left) of triple immunofluorescence for Pfkfb3 (red), Adgre1 (green) and nuclei (DAPI, blue), and quantification (right) of Pfkfb3 expression in the pulmonary macrophages of control or LPS-treated mice (*n* = 7). All data are represented as mean ± SEM, and ^***^P < 0.001 for indicated comparisons. Unpaired two-tailed Student's *t* test for **(A,D)** and one-way ANOVA with Bonferroni's *post-hoc* test for **(B,C)**.

Increased glycolytic flux has been shown in LPS-stimulated macrophages. We first analyzed the expression of glycolytic molecules in BMDMs treated with LPS for 4 h using genome mRNA expression profiling data (GSE125036) from the GEO database. As shown in Figure 1A, the expression of *Slc2a1, Hk1, Pfkfb3* and *Pfkp* was upregulated more than 2-fold in LPS-stimulated macrophages compared to untreated macrophages, and *Pfkfb3* is the highest expressed gene among the upregulated glycolytic genes. To further confirm the upregulation of *Pfkfb3* in LPS-stimulated macrophages, we first analyzed the mRNA levels of Pfkfb3 in LPS-stimulated BMDMs using real-time PCR, and found that LPS increased *Pfkfb3* mRNA expression in a time-dependent manner (Figure 1B). Consistently, the protein levels of Pfkfb3 were also significantly upregulated in BMDMs 2 h after LPS stimulation (Figure 1C). Moreover, increased Pfkfb3 expression in macrophages was also observed in lung from LPS-challenged mice compared with that from control mice (Figure 1D). Together, these findings suggested that inflammatory stimulator LPS reprograms the expression of Pfkfb3 in macrophages, and this may be a critical link in severe inflammatory responses in septic mice.

### Myeloid-Specific *Pfkfb3* Deficiency Protects Mice From LPS-Induced Cardiac Dysfunction

**Figure 2:**
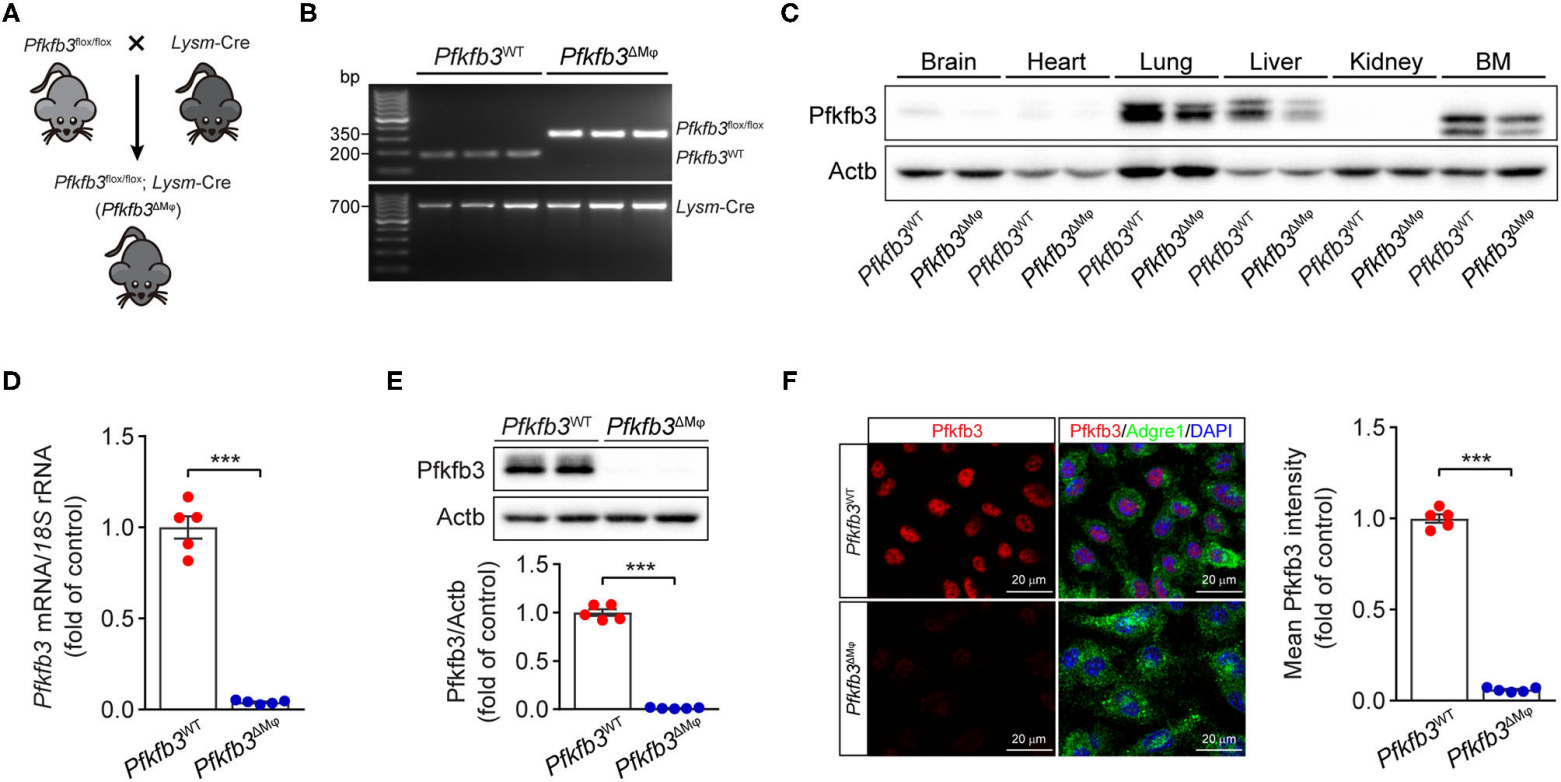
Generation and characterization of myeloid-specific *Pfkfb3*-deficient mice. **(A)** Diagram of the generation of myeloid-specific *Pfkfb3*-deficient mice (*Pfkfb3*^ΔMϕ^) by crossing *Pfkfb3*^flox/flox^ mice with *Lysm*-Cre mice. **(B)** Representative genotyping PCR results of *Pfkfb3*^WT^ mice and *Pfkfb3*^ΔMϕ^ mice. **(C)** Representative Western blot results of Pfkfb3 and Actb in bone marrow (BM) and main organs of *Pfkfb3*^WT^ and *Pfkfb3*^ΔMϕ^ mice. **(D)** qPCR analysis of *Pfkfb3* expression in BMDMs of *Pfkfb3*^WT^ mice and *Pfkfb3*^ΔMϕ^ mice (*n* = 5). **(E)** Representative Western blot results of Pfkfb3 and Actb in BMDMs of *Pfkfb3*^WT^ mice and *Pfkfb3*^ΔMϕ^ mice (top) and relative ratio of Pfkfb3/Actb (bottom) were quantitated by densitometric analysis of the corresponding Western blots (*n* = 5). **(F)** Representative images (left) of triple immunofluorescence for Pfkfb3 (red), Adgre1 (green) and nuclei (DAPI, blue), and quantification (right) of Pfkfb3 expression in BMDMs of *Pfkfb3*^WT^ mice and *Pfkfb3*^ΔMϕ^ mice (*n* = 5). All data are represented as mean ± SEM, ***P < 0.001 for *Pfkfb3*^WT^ vs. *Pfkfb3*^ΔMϕ^ (unpaired two-tailed Student's *t* test).

To determine the contribution of Pfkfb3-mediated glycolysis in macrophages to LPS-induced sepsis, we generated myeloid-specific *Pfkfb3* deficient mice (*Pfkfb3*^ΔMϕ^) and control mice (*Pfkfb3*^WT^) by intercrossing *Pfkfb3*^flox/flox^ mice with *Lysm*-Cre mice (Figures 2A,B). The protein level of Pfkfb3 was obvious decreased in main organs (including lung and liver) and bone marrow from *Pfkfb3*^ΔMϕ^ mice compared with those from *Pfkfb3*^WT^ mice (Figure 2C), indicating that Pfkfb3 is highly expressed in myeloid cells. The mRNA and protein analysis showed that Pfkfb3 expression was barely detectable in BMDMs cultured with bone marrow from *Pfkfb3*^ΔMϕ^ mice compared with those cultured with bone marrow of *Pfkfb3*^WT^ mice (Figures 2D–F), confirming effective deletion of Pfkfb3 in the myeloid cells of *Pfkfb3*^ΔMϕ^ mice.

**Figure 3:**
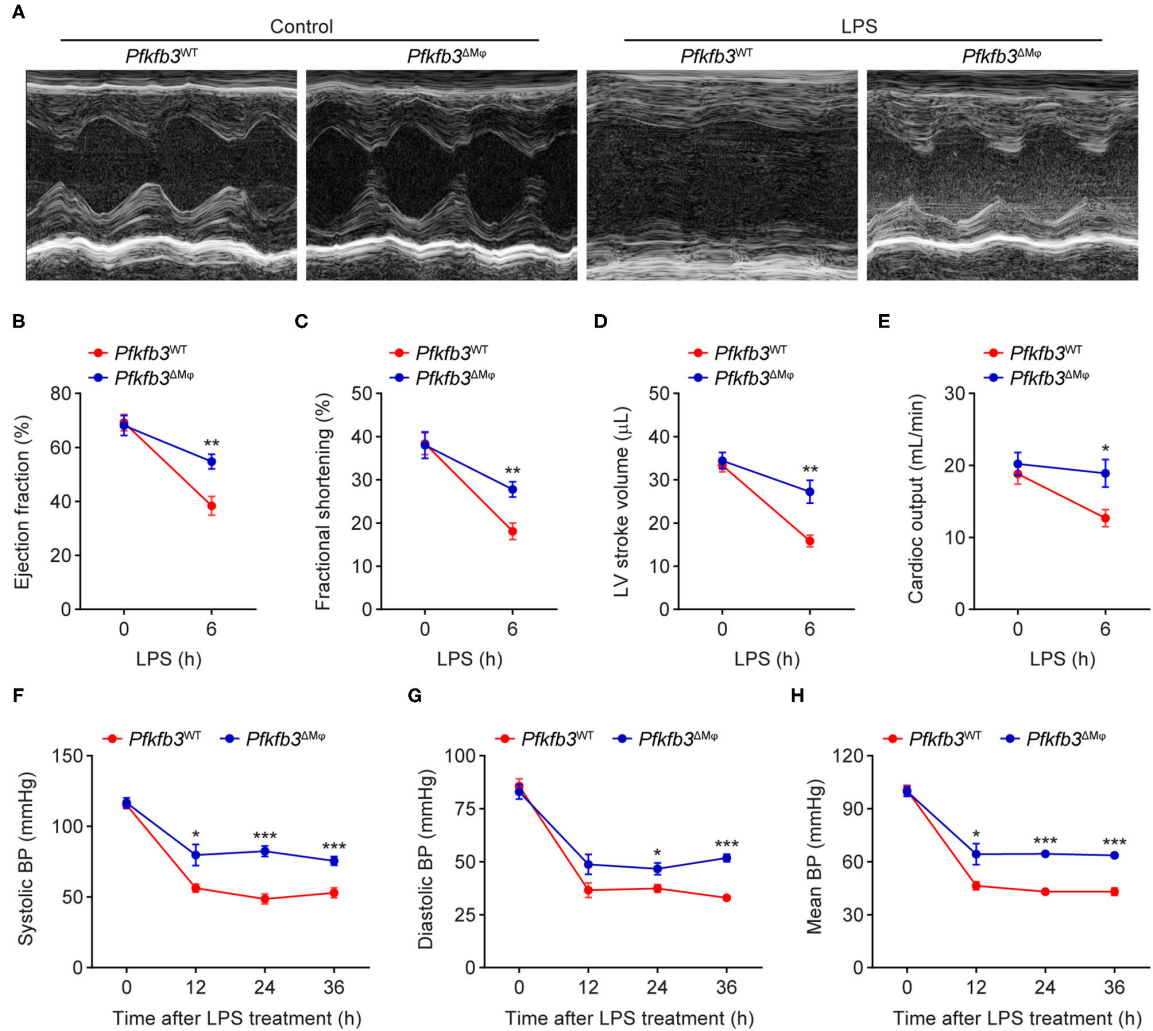
Myeloid-specific *Pfkfb3* deficiency protects mice from LPS-induced cardiac dysfunction. **(A)** Representative M-mode echocardiograms of *Pfkfb3*^WT^ mice and *Pfkfb3*^ΔMϕ^ mice 6 h after LPS injection. **(B-E)** Quantification of ejection fraction **(B)**, fractional shortening **(C)**, left ventricle stroke volume **(D)**, and cardiac output **(E)** of *Pfkfb3*^WT^ mice and *Pfkfb3*^ΔMϕ^ mice 6 h after LPS injection (*n* = 5). **(F–H)** Systolic blood pressure **(F)**, diastolic blood pressure **(G)**, and mean blood pressure **(H)** of *Pfkfb3*^WT^ mice and *Pfkfb3*^ΔMϕ^ mice before and after LPS injection (*n* = 8). All data are represented as mean ± SEM, *P < 0.05, **P < 0.01 and ***P < 0.001 for *Pfkfb3*^WT^ vs. *Pfkfb3*^ΔMϕ^ (unpaired two-tailed Student's *t* test).

To examine the role of myeloid Pfkfb3 in LPS-induced cardiac dysfunction (one of the features of multi-organ dysfunction in sepsis), *Pfkfb3*^WT^ and *Pfkfb3*^ΔMϕ^ mice were intraperitoneally administered with LPS, and cardiac assessment was used to record the cardiac parameters using M-mode echocardiography. As shown in Figures 3A–E, compared with the control mice, *Pfkfb3*^WT^ mice exhibited cardiac dysfunction 6 h after LPS challenge, as evidenced by decrease in ejection fraction, fractional shortening, left ventricle stroke volume and cardiac output. However, myeloid-specific *Pfkfb3* deficiency significantly prevented the decrease in cardiac function of *Pfkfb3*^WT^ mice observed after LPS injection (Figures 3A–E). Moreover, *Pfkfb3*^ΔMϕ^ mice exhibited significantly increased blood pressure compared to *Pfkfb3*^WT^ mice at 12 h after LPS injection, as indicated by increasing systolic blood pressure, diastolic blood pressure and mean blood pressure (Figures 3F–H). Together, these findings suggest that myeloid Pfkfb3 is a critical mediator in LPS-induced cardiac dysfunction *in vivo*.

### Myeloid-Specific *Pfkfb3* Deficiency Protects Mice From LPS-Induced Acute Lung Injury

**Figure 4:**
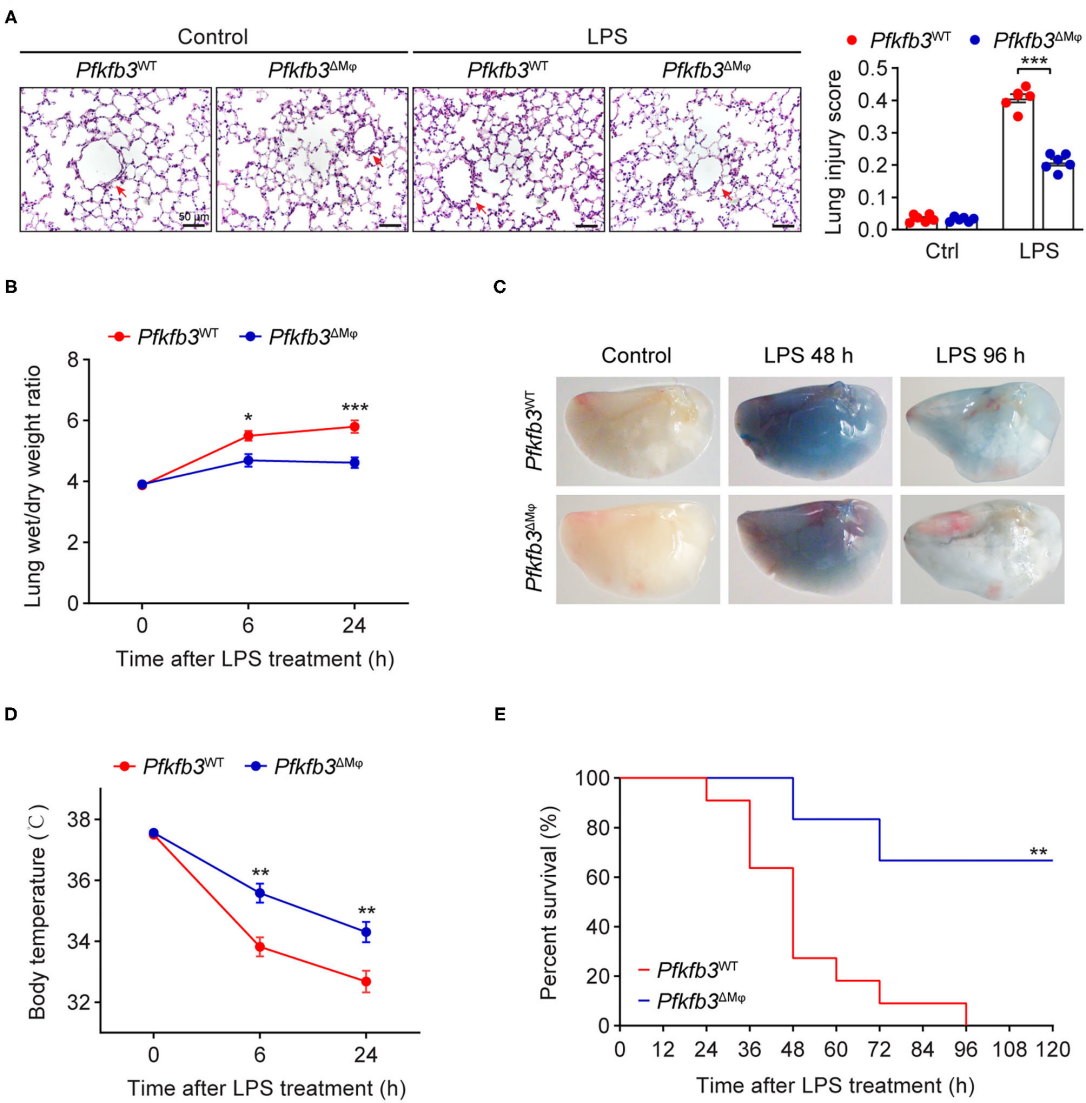
Myeloid-specific *Pfkfb3* deficiency protects mice from LPS-induced acute lung injury. **(A)** Representative images of hematoxylin and eosin staining (left) and lung injury score analysis (right) of lung sections from *Pfkfb3*^WT^ mice and *Pfkfb3*^ΔMϕ^ mice 6 h after LPS injection (*n* = 6). **(B)** The wet/dry weight ratio of lung tissue of *Pfkfb3*^WT^ mice and *Pfkfb3*^ΔMϕ^ mice after LPS injection (*n* = 6-8). **(C)** Representative images of Evans Blue staining of lung tissue of *Pfkfb3*^WT^ mice and *Pfkfb3*^ΔMϕ^ mice after LPS injection. **(D)** Changes of body temperature of *Pfkfb3*^WT^ mice and *Pfkfb3*^ΔMϕ^ mice after LPS injection (*n* = 9). **(E)** Survival curve of *Pfkfb3*^WT^ mice and *Pfkfb3*^ΔMϕ^ mice following LPS injection (*n* = 6–11). All data are represented as mean ± SEM, *P < 0.05, **P < 0.01 and ***P < 0.001 for indicated comparisons. Unpaired two-tailed Student's *t* test for **(B,D)** and Mantel-Cox test for **(E)**.

To investigate the effect of myeloid-specific *Pfkfb3* deficiency on LPS-induced acute lung injury, we first determined the histological alterations of lungs from LPS-challenged *Pfkfb3*^ΔMϕ^ and *Pfkfb3*^WT^ mice by hematoxylin and eosin (H&E) staining. The H&E staining results indicated a lower degree of recruitment of inflammatory cells into the lungs of *Pfkfb3*^ΔMϕ^ mice compared to those of *Pfkfb3*^WT^ mice (Figure 4A). To further examine the protective effect of myeloid-specific *Pfkfb3* deficiency on lung injury, the pulmonary permeability was assessed *in vivo*. The lung wet-to-dry ratio, which was used as an index to evaluate lung edema, was decreased in the lungs of *Pfkfb3*^ΔMϕ^ mice compared with those of *Pfkfb3*^WT^ mice after LPS injection (Figure 4B). Next, we injected Evans Blue in *Pfkfb3*^ΔMϕ^ and *Pfkfb3*^WT^ mice with or without LPS treatment. As shown in Figure 4C, Evans Blue leakage majorly decreased in the lungs of *Pfkfb3*^ΔMϕ^ mice compared to those of *Pfkfb3*^WT^ mice after LPS injection. In addition, *Pfkfb3*^ΔMϕ^ mice exhibited higher body temperature after LPS challenge compared to *Pfkfb3*^WT^ mice (Figure 4D). Moreover, we observed a marked improvement in mortality: 67% *Pfkfb3*^ΔMϕ^ mice survived after 96 h of the LPS-challenge whereas none of *Pfkfb3*^WT^ mice survived in the control group (Figure 4E). Collectively, these observations suggest that myeloid-specific *Pfkfb3* deficiency protects mice against LPS-induced lethality and tissue injury.

### Myeloid-Specific *Pfkfb3* Deficiency Attenuates LPS-Induced Inflammatory Responses in Mice

**Figure 5:**
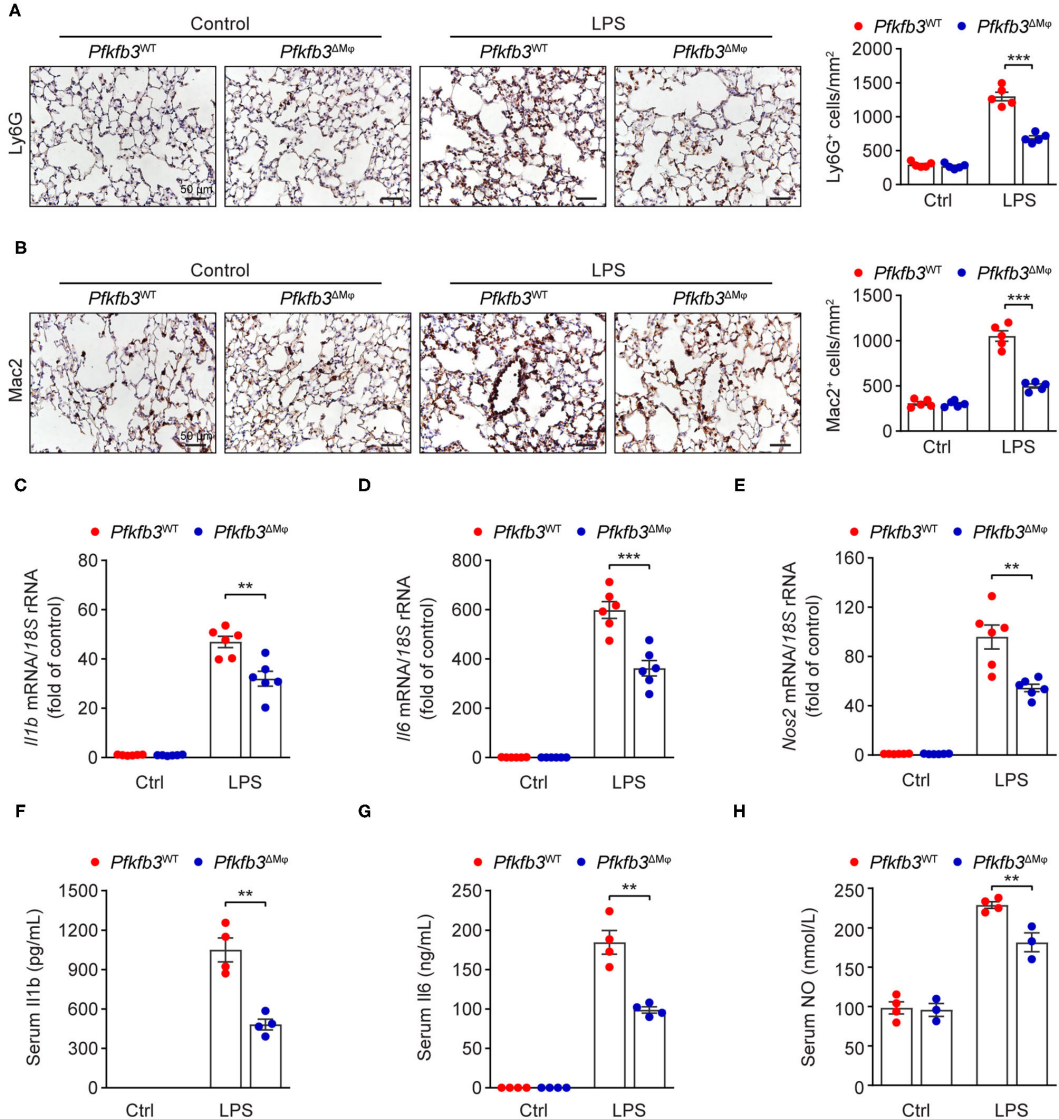
Myeloid-specific *Pfkfb3* deficiency attenuates LPS-induced inflammatory responses. **(A)** Representative images (left) and quantification (right) for immunohistochemical staining of the neutrophil marker Ly6G in lung sections of *Pfkfb3*^WT^ mice and *Pfkfb3*^ΔMϕ^ mice 6 h after LPS injection (*n* = 5). **(B)** Representative images (left) and quantification (right) for immunohistochemical staining of the macrophage marker Mac2 in lung sections of *Pfkfb3*^WT^ mice and *Pfkfb3*^ΔMϕ^ mice 6 h after LPS injection (*n* = 5). **(C–E)** qPCR analysis of the mRNA levels of *Il1b* **(C)**, *Il6* **(D)** and *Nos2* **(E)** in the lung of *Pfkfb3*^WT^ mice and *Pfkfb3*^ΔMϕ^ mice 6 h after LPS injection (*n* = 6). **(F,G)** ELISA analysis of Il1b **(F)** and Il6 **(G)** in serum of *Pfkfb3*^WT^ mice and *Pfkfb3*^ΔMϕ^ mice 6 h after LPS injection (*n* = 4). **(H)** NO levels in serum of *Pfkfb3*^WT^ mice and *Pfkfb3*^ΔMϕ^ mice 6 h after LPS injection (*n* = 3–4). All data are represented as mean ± SEM, **P < 0.01 and ***P < 0.001 for *Pfkfb3*^WT^ vs. *Pfkfb3*^ΔMϕ^ (unpaired two-tailed Student's *t* test).

Recruitment of myeloid cells into the lung represents a key feature of LPS-induced tissue injury. We first exposed *Pfkfb3*^ΔMϕ^ and *Pfkfb3*^WT^ mice to 12.5 mg/kg LPS for 6 h and then detected the infiltration of neutrophils and macrophages in the lung tissue with immunostaining. The results showed that the infiltration of Ly6G positive cells and Mac2 positive cells was significantly decreased in the lungs of *Pfkfb3*^ΔMϕ^ mice compared to those of *Pfkfb3*^WT^ mice (Figures 5A,B). Given that the recruitment of myeloid cells is mainly dependent on the excessive expression of proinflammatory mediators, including interleukin 1b (Il1b), interleukin 6 (Il6) and inducible nitric oxide synthase 2 (Nos2), we next analyzed the effect of myeloid-specific *Pfkfb3* deficiency on the expression of proinflammatory genes in lungs using real-time PCR. The mRNA levels of *Il1b, Il6* and *Nos2* were significantly increased in the lungs of LPS-challenged *Pfkfb3*^WT^ mice compared to those of control mice, whereas these mRNA levels were decreased in the lungs of LPS-challenged *Pfkfb3*^ΔMϕ^ mice compared to LPS-challenged *Pfkfb3*^WT^ mice (Figures 5C–E). Consistently, the serum levels of Il1b, Il6 and NO were significantly increased in LPS-challenged *Pfkfb3*^WT^ mice compared to control mice, whereas the serum levels of Il1b, Il6 and NO were decreased in LPS-challenged *Pfkfb3*^ΔMϕ^ mice compared to LPS-challenged *Pfkfb3*^WT^ mice (Figures 5F–H). Thus, these data suggest that myeloid-specific *Pfkfb3* deficiency protects mice from LPS-induced excessive inflammatory responses *in vivo*.

### Enhance Glycolysis in Macrophages Supports Their Proinflammatory Activity Upon LPS Stimulation

**Figure 6:**
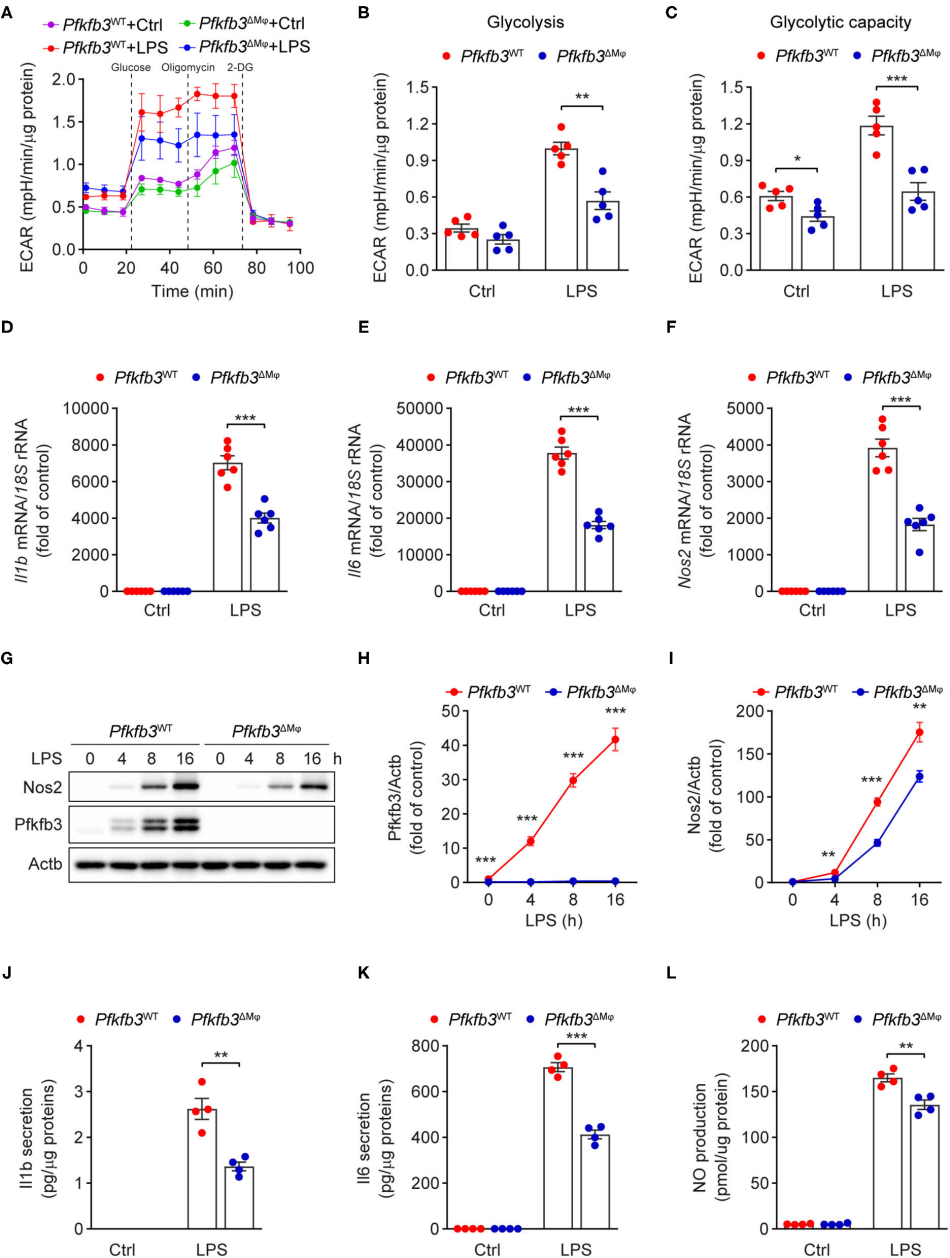
Enhance glycolysis in macrophages supports their proinflammatory activity upon LPS stimulation. **(A)** ECAR profile shows glycolytic function in *Pfkfb3*^WT^ and *Pfkfb3*^ΔMϕ^ BMDMs treated with LPS (100 ng/mL) for 6 h (*n* = 5). **(B,C)** Quantification of glycolysis [**(B)**, ECAR after glucose addition and subtracted by the average of basal values] and glycolytic capacity [**(C)**, ECAR after oligomycin addition and subtracted by the average of basal values] from **(A)** (*n* = 5). **(D–F)** qPCR analysis of the mRNA levels of *Il1b* **(D)**, *Il6* **(E)** and *Nos2* **(F)** in *Pfkfb3*^WT^ and *Pfkfb3*^ΔMϕ^ BMDMs treated with LPS (100 ng/mL) for 4 h (*n* = 6). **(G–I)** Representative Western blot results of Nos2, Pfkfb3 and Actb in *Pfkfb3*^WT^ and *Pfkfb3*^ΔMϕ^ BMDMs treated with LPS (100 ng/mL) for the indicated times **(G)** and relative ratio of Pfkfb3/Actb **(H)** and Nos2/Actb **(I)** were quantitated by densitometric analysis of the corresponding Western blots (*n* = 4). **(J,K)** ELISA analysis of Il1b **(J)** and Il6 **(K)** secretion in the supernatants of *Pfkfb3*^WT^ and *Pfkfb3*^ΔMϕ^ BMDMs treated with LPS (100 ng/mL) for 16 h (*n* = 4). **(L)** Quantification of NO secretion in the supernatants of *Pfkfb3*^WT^ and *Pfkfb3*^ΔMϕ^ BMDMs treated with LPS (100 ng/mL) for 16 h (*n* = 4). All data are represented as mean ± SEM, **P < 0.01 and ***P < 0.001 for *Pfkfb3*^WT^ vs. *Pfkfb3*^ΔMϕ^ (unpaired two-tailed Student's *t* test).

Having observed that myeloid Pfkfb3 deficiency reduced LPS-induced inflammation *in vivo*, we further investigated whether Pfkfb3 regulates the expression of proinflammatory cytokines and *Nos2* in macrophages *in vitro*. We first analyzed the glycolytic flux in BMDMs cultured with bone marrow from *Pfkfb3*^ΔMϕ^ mice or corresponding wild-type *Pfkfb3*^WT^ mice using the Seahorse Extracellular Flux analyzer. As shown in Figures 6A–C, *Pfkfb3*^ΔMϕ^ BMDMs displayed significantly reduced glycolysis and glycolysis capacity in response to LPS compared to *Pfkfb3*^WT^ BMDMs. Importantly, *Pfkfb3*^ΔMϕ^ BMDMs displayed reduced mRNA levels of *Il1b, Il6* and *Nos2* in response to LPS compared with *Pfkfb3*^WT^ BMDMs (Figures 6D–F). Consistent with mRNA levels, *Pfkfb3*^ΔMϕ^ BMDMs displayed significantly reduced protein levels of Nos2 in response to LPS compared with *Pfkfb3*^WT^ BMDMs (Figures 6G–I). Moreover, the secretion of Il1b, Il6 and NO was significantly decreased in *Pfkfb3*^ΔMϕ^ BMDMs compared to that in *Pfkfb3*^WT^ BMDMs (Figures 6J–L). Thus, these data suggest that inhibition of glycolysis with *Pfkfb3* deletion in macrophages reduces LPS-induced expression of proinflammatory cytokines and Nos2 *in vitro*.

### Pfkfb3 Inactivation in Macrophages Suppresses LPS-Induced Inflammation *via* the NF-κB Signaling Pathway

**Figure 7:**
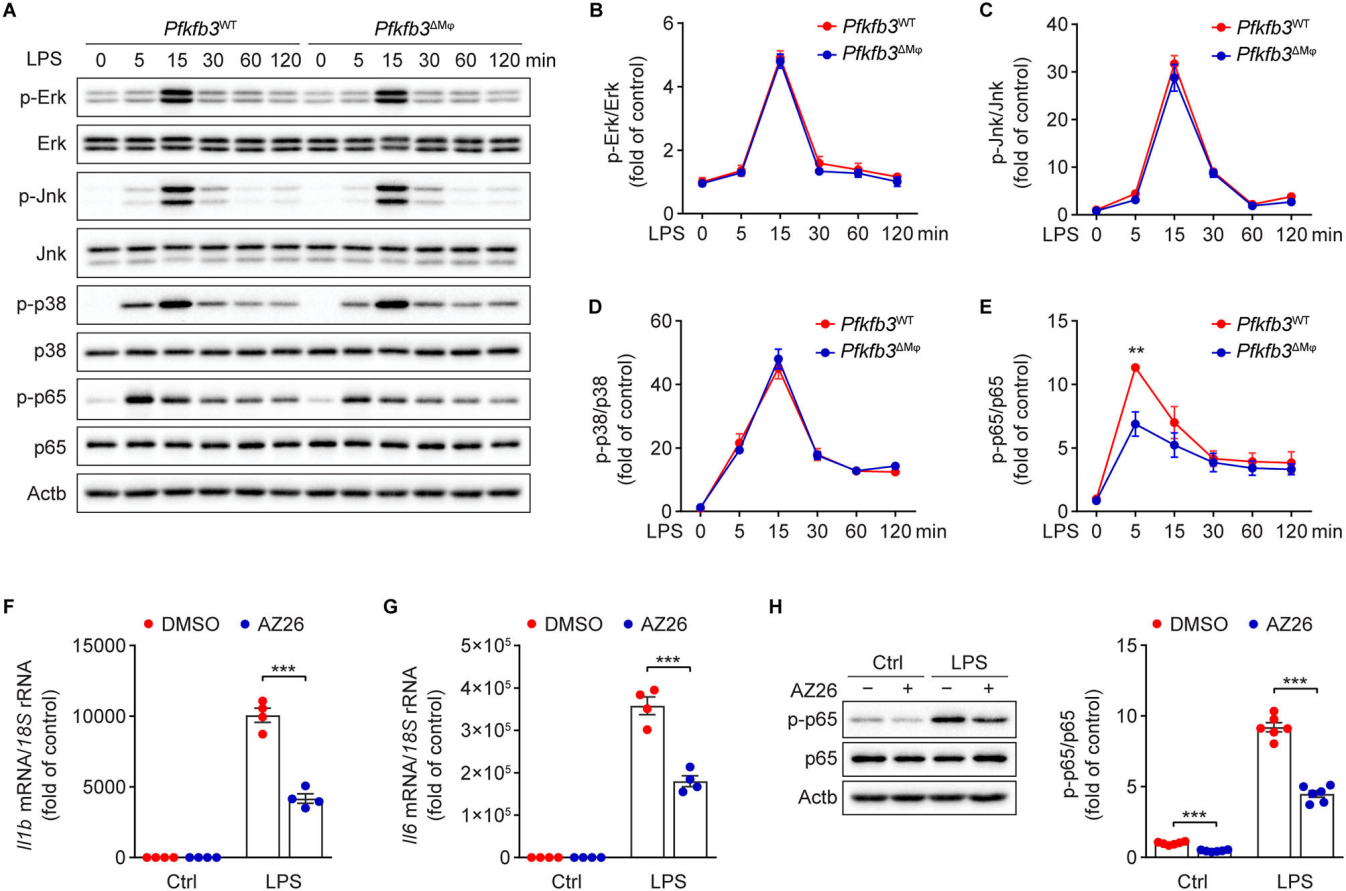
Pfkfb3 inactivation in macrophages suppresses LPS-induced inflammation via the NF-κB signaling pathway. **(A–E)** Representative Western blot results **(A)** of phosphorylated and total level of Erk, Jnk, p38 and p65 in *Pfkfb3*^WT^ and *Pfkfb3*^ΔMϕ^ BMDMs treated with LPS (100 ng/mL) at the indicated time points, and relative ratio of p-Erk/Erk **(B)**, p-Jnk/Jnk **(C)**, p-p38/p38 **(D)** and p-p65/p65 **(E)** were quantitated by densitometric analysis of the corresponding Western blots (*n* = 4). **(F,G)** qPCR analysis of the mRNA levels of *Il1b* **(F)** and *Il6* **(G)** in BMDMs treated with LPS (100 ng/mL) for 4 h, with or without AZ26 (10 μM) pretreatment for 30 min (*n* = 4). **(H)** Representative Western blot results (left) of p-p65, p65 and Actb in BMDMs treated with LPS (100 ng/mL) for 5 min, with or without AZ26 (10 μM) pretreatment for 30 min, and relative ratio of p-p65/p65 (right) was quantitated by densitometric analysis of the corresponding Western blots (*n* = 4). All data are represented as mean ± SEM, **P < 0.01 and *** *P* < 0.001 for indicated comparisons (unpaired two-tailed Student's *t* test).

We next investigated the molecular mechanisms by which LPS-induced signal transduction is regulated by Pfkfb3 in macrophages. The activation of nuclear factor-κB (NF-κB) and mitogen-activated protein kinase (MAPK) by LPS is the key signaling pathways for the induced expression of proinflammatory genes in macrophages. We analyzed the phosphorylation of Erk, Jnk, p38 and p65 in LPS-stimulated *Pfkfb3*^ΔMϕ^ and *Pfkfb3*^WT^ BMDMs by Western blot. As shown in Figures 7A–E, the protein levels of p-Erk, p-Jnk, p-p38 and p-p65 were markedly increased in *Pfkfb3*^WT^ BMDMs stimulated with LPS. However, of the above observed signaling molecule changes, only the upregulation of p-p65 by LPS was suppressed in *Pfkfb3*^ΔMϕ^ BMDMs treated with LPS. Moreover, similar to *Pfkfb3* knockout, AZ26, which is an inhibitor that abolishes the enzyme activation of Pfkfb3 (21), could antagonize the upregulation of proinflammatory cytokine *Il1b* and *Il6* expression in LPS-treated wild type BMDMs (Figures 7F,G). Furthermore, the phosphorylation of p65 was also antagonized by AZ26 in LPS-treated BMDMs (Figure 7H). These data suggest that the NF-κB signaling pathway is involved in the downregulation of proinflammatory mediators in macrophages by Pfkfb3 inactivation.

## Discussion

In the present study, we demonstrated the effect of myeloid Pfkfb3 in LPS-induced sepsis in mice. Deletion of myeloid *Pfkfb3* reduces LPS-induced sepsis and inflammatory responses by decreasing lung infiltration of macrophages and neutrophils and improving lung edema, cardiac dysfunction and hypotension. This improved phenotype is associated with the decrease in the expression of Nos2 and inflammatory cytokines in macrophages via decreased NF-κB signaling with deficiency/inhibition of Pfkfb3.

Enhanced glycolytic flux has been recognized as a metabolic signature of activated macrophages (8, 9, 22). Increased glycolysis enables macrophages to rapidly generate ATP and biosynthetic intermediates to support proinflammatory cytokine production (23–25). Extensive studies have reported the effect of glycolytic molecules in regulation of macrophage activation (26–29). PFKFB3, an isoform of 6-phosphofructo-2-kinase/fructose-2,6-bisphosphatase (PFK-2), regulates intracellular levels of the glycolytic intermediate fructose-2,6-bisphosphate (F-2,6-BP), which allosterically activates the second rate-limiting enzyme 6-phosphofructo-1-kinase (PFK-1), leading to increase glycolytic flux (30). However, the effect of myeloid Pfkfb3 on modulation of LPS-induced sepsis has not been studied yet. RNA-Seq data reveals that macrophage *Pfkfb3* was the most significantly upregulated glycolytic gene in response to LPS treatment (10). In addition, *PFKFB3* was also upregulated in myeloid blood cells from critically ill COVID-19 patients (31). It has been reported that *Pfkfb3* is a hypoxia-inducible gene that is stimulated through HIF interaction with the consensus HRE site in its promoter region (32). Therefore, increased Pfkfb3 expression in macrophages may be attributed to an enhanced HIF pathway upon proinflammatory stimuli. Recent study has shown that p65 binds to the PFKFB3 promoter in response to treatment with proinflammatory cytokine in endothelial cells (33), thus, upregulation of Pfkfb3 in macrophages may also be due to activated NF-κB signaling under LPS stimuli.

Myeloid Pfkfb3 knockout-decreased macrophage inflammatory response protects mice from LPS-induced sepsis. It has been well accepted that inhibited macrophage glycolysis protects mice from LPS-induced sepsis (13, 26). For example, inactivation of hexokinase 1 (HK1), the first rate-limiting enzyme of the glycolytic pathway, suppressed macrophage proinflammatory responses and LPS-induced sepsis in mice (26, 27). This has been associated with reduced Il1b production in macrophages and reduced serum levels of Tnfa and nitric oxide (NO). Moreover, knockout of myeloid pyruvate kinase M2 (Pkm2), the final rate-limiting enzyme in glycolysis, also reduced septic death in mice (29). In the current study, LPS-induced septic death and organ dysfunction including pulmonary edema and cardiac dysfunction were decreased in *Pfkfb3*^ΔMϕ^ mice. Decreased serum levels of NO and proinflammatory cytokines, and reduced infiltration of macrophages in lung tissue also occurred in LPS-treated *Pfkfb3*^ΔMϕ^ mice. A further *in vitro* study demonstrated increased glycolytic flux induced by LPS was significantly dampened in *Pfkfb3*^ΔMϕ^ BMDMs, indicating LPS-induced Pfkfb3 expression is a critical step in the induction of glycolysis in activated macrophages. LPS-induced upregulation of Nos2 and proinflammatory cytokine Il1b and Il6 was also decreased in *Pfkfb3*^ΔMϕ^ BMDMs. Therefore, our findings on decreased septic death in LPS-treated *Pfkfb3*^ΔMϕ^ mice, at least in part, are associated with reduced macrophage activation through decreased the expression of Nos2 and inflammatory cytokines such as Il1b and Il6 (34–37).

Pfkfb3 deficiency/inhibition reduces macrophage proinflammatory responses through the NF-κB signaling pathway. Genetic and pharmacological inhibition of macrophage Pfkfb3 decreased LPS-induced proinflammatory gene expression. This is due to decreased NF-κB activity which is evidenced by the decreased level of p65 phosphorylation (38). The decreased level of p65 phosphorylation in this study is in line with previous studies in which the decreased level of p65 phosphorylation without affecting MAPK signaling pathway in human monocytes upon proinflammatory stimuli (39, 40). This observation is also in agreement with several recent studies in which *PFKFB3* knockdown in endothelial cells decreases proinflammatory stimuli-induced proinflammatory responses through downregulation of p65 phosphorylation (41–43). The underlying mechanisms by which Pfkfb3 deficiency directly controls the inactivation of NF-κB signaling may include the following possibilities. First, most of the downstream signaling processes consume ATP during activating NF-κB signaling in response to LPS. For example, LPS-induced NF-κB activation is dependent on the ATP-dependent proteolysis of pIκBα (44). Given the fact that LPS-stimulated macrophages predominantly use glycolysis to generate ATP (45), it is likely that decreased glycolytic ATP production in *Pfkfb3* knockout macrophages reduces ATP-dependent NF-κB activation after LPS stimulation. Second, LPS-driven glycolytic ATP production facilitates an increase in mitochondrial membrane potential that is required for the generation of reactive oxygen species (ROS) (46). The decreased glycolytic flux upon LPS stimulation in *Pfkfb3*-deficient macrophages may result in reduced mitochondrial ROS production, which decreases activation of the redox-sensitive NF-κB (47). Finally, a recent study shows that lactate, a byproduct of glycolysis, enhances NF-κB activity and the expression of IL1B and IL6 in macrophages (48). The data from us has shown that *Pfkfb3* knockout reduced glycolytic flux upon LPS stimulation in BMDMs, which may also be responsible for downregulated NF-κB activation in *Pfkfb3*^ΔMϕ^ BMDMs.

Other possible mechanisms, such as the epigenetic effects of myeloid *Pfkfb3* deficiency, may also contribute to the alleviated macrophage activation and systemic inflammatory responses in LPS-treated *Pfkfb3*^ΔMϕ^ mice. Our recent study has shown that the deletion of *Pfkfb3* in macrophages reduces proinflammatory gene induction through downregulated hyperglycolysis-mediated histone acetylation (28). Therefore, it is very likely that decreased hyperglycolysis-mediated histone acetylation in *Pfkfb3*-deficient macrophages upon LPS stimulation contributes to the decreased proinflammatory gene expression in septic mice observed in the current study (49, 50).

In summary, our findings demonstrate that inactivation of macrophage Pfkfb3 can reduce LPS-induced organ dysfunction and death *via* decreased macrophage glycolysis and proinflammatory gene induction. A recent study has shown that inhibition of PFKFB3 hampers the progression of atherosclerosis and promotes plaque stability mainly through decreased glycolysis in monocytes and macrophages (51). Although further study is required to identify the role of PFKFB3 in other types of cells in the regulation of inflammatory diseases, it is very likely that regulation of macrophage PFKFB3 is a potential therapeutic strategy for the treatment of inflammatory diseases such as sepsis and atherosclerosis.

## Limitation

This study had some limitations. First, in addition to reduced lactate production, *Pfkfb3* deficiency in activated macrophages may change glycolysis-linked pathways such as pentose phosphate, glycogenesis, hexosamine, *de novo* serine synthesis and tricarboxylic acid cycle. Each of the above flux pathways is an important regulator in inflammatory response. Assessing these pathways by glucose flux with ^13^C-glucose tracer in activated *Pfkfb3*^WT^ and *Pfkfb3*^ΔMϕ^ BMDMs will be needed in future study. Second, as macrophages are critical in the host response to infection, thereby, it remains unclear whether blocking of macrophage glycolysis and cytokine production by deletion of *Pfkfb3* would impair the role of macrophages in bacterial clearance and resolution of infection-mediated sepsis. An infection-induced sepsis model is required in future study. Third, murine system *in vitro* and *in vivo* is solely used in this study. A difference in glucose metabolism between human and murine system has been reported (52, 53). Thus, the role of PFKFB3 in inflammatory and metabolic response in human macrophages need to be determined in future study.

## Data Availability Statement

The raw data supporting the conclusions of this article will be made available by the authors, without undue reservation.

## Ethics Statement

The animal study was reviewed and approved by the Institutional Animal Care and Use Committee of Augusta University.

## Author Contributions

JX, LW, QY, YS, ZB, RL, ZL, MH, KO, and YH designed the research. JX, LW, QY, QM, YZ, YC, XM, and QD performed experiments. JX, LW, QY, and YH analyzed data. JX, TL, ZL, MH, KO, and YH wrote and revised the manuscript. YS, ZB, RL, ZL, MH, KO, and YH provided the reagents or materials and participated in experimental design. YH had primary responsibility for the final content. All authors read and approved the final manuscript.

## Funding

This work was supported in part or in whole by grants from National Science Foundation of China Grants 82100506, 81870324, and 82070461; China Postdoctoral Science Foundation 2020M680003 and 2020M670051; Guangdong Basic and Applied Basic Research Foundation 2020A1515010010 and 2019A1515110111; Shenzhen Science and Technology Innovation Committee Grants JCYJ20190808155605447, JCYJ20170810163238384, JCYJ20190808155801648, JCYJ201704121-50405310, and GXWD20201231165807007-20200818123312001. Shenzhen-Hong Kong Institute of Brain Science-Shenzhen Fundamental Research Institutions 2019SHIBS0004; American Heart Association Grant 19TPA34910043. National Institutes of Health Grants R01HL134934, R01EY030500, R01HL142097, and R01HL138410. VA Merit Review Grant BX002035.

## Conflict of Interest

The authors declare that the research was conducted in the absence of any commercial or financial relationships that could be construed as a potential conflict of interest.

## Publisher's Note

All claims expressed in this article are solely those of the authors and do not necessarily represent those of their affiliated organizations, or those of the publisher, the editors and the reviewers. Any product that may be evaluated in this article, or claim that may be made by its manufacturer, is not guaranteed or endorsed by the publisher.
