# Supplementary material for: Deficiency of Myeloid Pfkfb3 Protects Mice From Lung Edema and Cardiac Dysfunction in LPS-Induced Endotoxemia
Source: Front Cardiovasc Med. 2021 Sep 29;8:745810. doi: 10.3389/fcvm.2021.745810 (PMC8511447; doi:10.3389/fcvm.2021.745810)
Supplement: Supplementary file 1 [file Table_1.DOCX]

**Supplementary Table 1. Mouse primer sequences for real-time RT-PCR analyses.**

| Gene | Forward primer (5′-3′) | Reverse primer (5′-3′) |
| --- | --- | --- |
| *Pfkfb3* | GATCTGGGTGCCCGTCGATCACCG | CAGTTGAGGTAGCGAGTCAGCTT |
| *Il1b* | TGTCTTGGCCGAGGACTAAGG | TGGGCTGGACTGTTTCTAATGC |
| *Il6* | GTTCTCTGGGAAATCGTGGA | TGTACTCCAGGTAGCTATGG |
| *Nos2* | CAGCTGGGCTGTACAAACCTT | CATTGGAAGTGAAGCGTTTCG |
| *18S* rRNA | CTTAGAGGGACAAGTGGCG | ACGCTGAGCCAGTCAGTGTA |
